# 

**Published:** 1904-12

**Authors:** 


ESTABLISHED IN 1839

THE OLDEST DENTAL MAGAZINE IN THE WORLD
INDEPENDENT---------PUBLISHED BY DENTISTS FOR DENTISTS

VOLUME XXXV NUMBER 12 — THIRD SERIES — DEC. 1904

EDITOR:
WM. GIRD BEECROFT, D. D. S., Madison, Wis.
ASSOCIATE EDITORS:
ALBERT H. KETCHAM, D. D. S., Denver, Colo.
EMORY A. BRYANT, D. D. S., Washington, D. C.
GEO. S MARTIN, D. D. S., L. D. S Toronto Junction, Ontario.

Published on the 10th day of each month.
Subscription Price, $1.00 per year.
Foreign Countries, $1.50 per year.
Address all communications, both business and editoria to the editor.
Entered as Second Class Matter, Madison, Wis.

